# Should we measure intra-abdominal pressures in every intensive care patient?

**DOI:** 10.1186/2110-5820-2-S1-S9

**Published:** 2012-07-05

**Authors:** Joel Starkopf, Kadri Tamme, Annika Reintam Blaser

**Affiliations:** 1 Department of Anaesthesiology and Intensive Care, University of Tartu, 8 L. Puusepa Str, 51014, Tartu, Estonia; 2 Clinic of Anaesthesiology and Intensive Care, Tartu University Hospital, 8 L. Puusepa Str, 51014, Tartu, Estonia; 3 Department of Intensive Care Medicine, University Hospital (Inselspital) and University of Bern, 3010 Bern, Switzerland

**Keywords:** intra-abdominal pressure, intra-abdominal hypertension, abdominal compartment syndrome, patient monitoring, intensive care, epidemiology.

## Abstract

Intra-abdominal pressure (IAP) is seldom measured by default in intensive care patients. This review summarises the current evidence on the prevalence and risk factors of intra-abdominal hypertension (IAH) to assist the decision-making for IAP monitoring.

IAH occurs in 20% to 40% of intensive care patients. High body mass index (BMI), abdominal surgery, liver dysfunction/ascites, hypotension/vasoactive therapy, respiratory failure and excessive fluid balance are risk factors of IAH in the general ICU population. IAP monitoring is strongly supported in mechanically ventilated patients with severe burns, severe trauma, severe acute pancreatitis, liver failure or ruptured aortic aneurysms. The risk of developing IAH is minimal in mechanically ventilated patients with positive end-expiratory pressure < 10 cmH_2_O, PaO_2_/FiO_2 _> 300, and BMI < 30 and without pancreatitis, hepatic failure/cirrhosis with ascites, gastrointestinal bleeding or laparotomy and the use of vasopressors/inotropes on admission. In these patients, omitting IAP measurements might be considered.

In conclusions, clear guidelines to select the patients in whom IAP measurements should be performed cannot be given at present. In addition to IAP measurements in at-risk patients, a clinical assessment of the signs of IAH should be a part of every ICU patient's bedside evaluation, leading to prompt IAP monitoring in case of the slightest suspicion of IAH development.

## Review

Intra-abdominal hypertension (IAH) and abdominal compartment syndrome (ACS) contribute significantly to multiorgan failure in critically ill patients [1,2] and are associated with considerable morbidity and mortality [3-5]. Prevention is the most effective way to avoid the deleterious effects of IAH; therefore, recognising the risk factors and clinical signs of IAH is particularly important for improving intensive care outcomes. Common physiological parameters, such as blood pressure, electrocardiogram, heart rate and haemoglobin saturation, are routinely monitored in every patient in intensive care. In contrast, measurements of intra-abdominal pressure (IAP) are seldom used as a standard element of monitoring. Rather, it is quite common to measure IAP only when either a particular risk factor or the presence of IAH is recognised. Malbrain et al. suggest that the simple procedure of IAP monitoring is necessary in all patients at risk of IAH [6]. The questions remain how exactly to identify these at-risk patients, and whether our risk assessment is precise enough. Many causal and predisposing factors are listed in the consensus paper from the World Society of Abdominal Compartment Syndrome (WSACS) (http://www.wsacs.org) [7]. This long list, mainly based on pathophysiologic considerations and supported by only weak evidence, is difficult to apply at bedside.

The aim of this review is to summarise the current evidence on the prevalence and risk factors of IAH to assist the decision-making for IAP monitoring. Relevant articles and published reviews were identified and analysed through a PubMed search of English-language literature. The keywords 'intra-abdominal pressure', 'intra-abdominal hypertension' and/or 'abdominal compartment syndrome' were used. Special attention was paid to publications on the epidemiology and risk factors of IAH.

## Prevalence, incidence and risk factors of IAH/ACS in mixed ICU populations

Much of our initial knowledge about IAH and ACS originates from patients with specific conditions such as ruptured aortic aneurysms, abdominal trauma, severe burns and severe acute pancreatitis [8-11]. These diagnostic groups, however, account for a minority of admissions in most ICUs. Although we have learned much about the pathophysiology and treatment of IAH from these studies, the true prevalence, impact on outcome, and risk factors of IAH remain to be clarified. To avoid bias from the preselection of patients, one should seek studies where all consecutive ICU admissions, independent of diagnosis, length of stay, mode of ventilation, etc. were included and the epidemiology of IAH addressed. We are not aware of any study meeting these criteria. The data on epidemiology presented below are, therefore, based on extrapolations from those studies that most resembled these ideal criteria and measured IAP in the general ICU populations.

The results of relevant studies are summarised in Table 1[6]. The studies on IAH are directly comparable only since 2006, when consensus definitions were published [7]. Two papers from 2004 and 2005, however, are so far the only multicentre studies; they used almost the same definitions as later in WSACS consensus statement and are, therefore, included in the analysis. The original definition of IAH, sustained or repeated IAP ≥ 12 mmHg, is not always easy to apply because intermittent measurements without fixed intervals are mostly used in practice. Therefore, a definition of IAH of the mean IAP ≥ 12 mmHg on one day was used in parallel.

**Table 1:** Prevalence, incidence and risk factors of IAH in mixed ICU populations

*Authors, year*	*Study type*	*Study period*	*Inclusion criteria*	*Patients included*	*Patients treated in study unit(s) during the study period *	*IAH threshold*	*IAH prevalence on admission day*	*IAH incidence*	*Independent risk factors of IAH *
Malbrain^3^, 2004	Multi-centre, 1-day point prevalence	1 day	Adults, ICU stay > 24 h	97	NA	Max IAP ≥ 12 mmHg Mean IAP ≥ 12 mmHg	58.8% (study day) 23.7%	NA	- BMI
Malbrain^4^, 2005	Prospective, Multi-centre	4 weeks	Adults, ICU stay > 24 h	265	NA	Mean IAP > 12 mmHg	32.1%	56% during 1^st ^ICU week	- Liver dysfunction - Abdominal surgery - Fluid resuscitation - Ileus
Vidal^12^, 2008	Prospective, single-centre	8 months	Adults, expected to stay > 24 h	83	153	IAP ≥ 12 mmHg in at least three consecutive measurements	23.0%	54.0% during 1^st ^ICU week	- Fluid resuscitation - Acidosis - Hypotension - Gastroparesis/ileus - ARDS - Mechanical ventilation
Dalfino^13^, 2008	Prospective, single-centre	6 months	Adults, ICU stay > 24 h	123	215	IAP ≥ 12 mmHg in at least two consecutive measurements	19.0%	31.0% during ICU stay	- Age - Cumulative fluid balance - Shock
Reintam^14^, 2008	Prospective, single-centre	24 months	Adults, mechanical ventilation + one additional predisposing condition for IAH	257	754	Sustained or repeated IAP ≥ 12 mmHg	23.3%	37.0% during ICU stay	- No independent risk factors identified
Reintam Blaser^15^, 2011	Prospective, single-centre	33 months	Mechanically ventilated adults, expected to stay > 24 h	563	922	Sustained or repeated IAP ≥ 12 mmHg	20.4%	32.3% during ICU stay	- Pancreatitis - Hepatic failure/cirrhosis with ascites - GI bleeding - PEEP > 10 cmH_2_O - Vasopressor/inotrope - BMI > 30 kg/m^2^ - Laparotomy - PaO_2_/FiO_2 _< 300 mmHg

In 2004, the first study of IAH epidemiology in a mixed ICU population was performed. In it, 97 patients treated in 13 ICUs in six countries for at least 24 h were enrolled for 1-day point-prevalence assessments. IAH, defined as a maximum IAP ≥ 12 mmHg, was observed in 58.8% of cases [3], whereas defining IAH according to the mean IAP resulted in a prevalence of 23.7%. A second multicentre study included 265 consecutive patients (mean Acute Physiology and Chronic Health Evaluation II score (APACHE) II score 17.4; 46.8% of the medical profile) who stayed in the ICU for at least 24 h [4]. IAH, defined as the mean IAP > 12 mmHg, was observed in 32.1% and ACS in 4.2% of these patients on admission, whereas the development of IAH during the ICU stay was an independent predictor of mortality. Independent predictors of IAH were liver dysfunction (defined as decompensated or compensated cirrhosis or other liver failure with ascites), abdominal surgery (with or without laparoscopy, reduction of a hernia, tight closure, or abdominal banding with a postoperative Velcro belt to prevent incisional hernia), massive fluid resuscitation (defined as 3.5 L of colloids or crystalloids in the 24 h before the study) and ileus. At the same time, there was no difference in the prevalence of IAH between medical and surgical patients.

Vidal et al. collected prospective data on 83 critically ill patients staying in an ICU for at least 24 h (mean APACHE II 19; 47% of the medical profile) [12]. Considering mean IAP, 23% of patients had IAH on admission, but another 31% developed it afterwards. Twelve per cent of patients suffered from ACS. Patients with IAH were sicker and had a higher mortality rate, whereas the maximum IAP was identified as an independent predictor of mortality.

Dalfino et al. followed 123 consecutive patients staying in an ICU for at least 24 h, and they observed a 30.1% incidence of IAH [13]. The raw hospital mortality rate was significantly higher in patients with IAH; however, risk-adjusted rates were not different. IAH was significantly associated with age, cumulative fluid balance, shock, sepsis and abdominal surgery, whereas only the first three were independent risk factors.

In a study of 264 mechanically ventilated patients who presented at least one additional predisposing factor associated with IAH (admission due to severe trauma, abdominal surgery, pancreatitis or post-cardiopulmonary resuscitation status; fluid resuscitation above 5 L/24 h; vasoactive or inotropic support; or renal replacement therapy), the development of IAH was identified as an independent risk factor of death [14]. The incidence of IAH was 37.0%. The patients with IAH had higher age, BMI, fluid gain and disease severity scores during the admission day in the univariate analysis, whereas independent risk factors for IAH were not defined.

In the largest study so far, Reintam Blaser et al. investigated 563 out of 635 consecutive patients who were mechanically ventilated and stayed over 24 h in the ICU. During the entire ICU period, IAH occurred in 32.3% of patients [15]. ACS developed in six patients (1.1%). A checklist was developed to facilitate the decisions for IAP monitoring at bedside. However, the single-centre design of the study and the fact that not all consecutive patients were studied limit its practical value.

The preliminary results of a multicentre study analysing 358 patients from 39 ICUs, which included consecutive patients requiring mechanical ventilation for at least 6 h [16], assessed the validity of predisposing conditions of IAH proposed by the consensus statement [7]. This study found that considerable proportion of patients (22.0%) without any aetiological factor other than mechanical ventilation still developed IAH. Furthermore, among the patients presenting five or more predisposing factors, more than one-third did not develop IAH.

In summary, IAH, depending on the sample population, may occur in 20% to 40% of intensive care patients. The incidence of ACS in general cohorts is 5% to 10% in earlier reports but considerably less in recent studies. This fact leads us to speculate that awareness of IAH, and its proper medical treatment may have reduced the incidence of progression from IAH to ACS. Importantly, no study has assessed the incidence of IAH in all consecutive admissions to the ICU. The studies that included patients who were expected to stay for at least 24 h in the ICU must certainly have missed some patients who actually stayed for more than 24 h. Therefore, the exact incidence of IAH/ACS in mixed ICU patient populations remains unclear.

### IAH/ACS in specific patient groups

IAH/ACS has frequently been described in *severe trauma patients*. The incidence of ACS in trauma patients undergoing emergency laparotomy/damage-control surgery ranges from 5% to 14% depending on the severity of the insult and the extent of abdominal packing applied [9,17]. The liberal use of open-abdomen techniques has decreased the incidence of ACS in these patients [18]; however, monitoring IAP is still mandatory, as ACS can develop in cases of open abdomen [19,20]. IAH/ACS may also be observed in patients without abdominal injuries and is then associated with severe shock that requires aggressive fluid resuscitation; the incidence of IAH/ACS is 0.07% of all trauma admissions and 9% of shock trauma patients [21]. Among all trauma victims admitted to ICUs, IAP above 20 mmHg can be observed in 2% of cases [22]. Thus, severe trauma, especially when abdominal injuries are involved, carries a considerable risk of IAH and ACS.

IAH/ACS is also reported in a number of small series of *severely burned patients *[23,24] and is currently considered a life-threatening complication of thermal injury [25]. Extensive swelling and ascites due to profound systemic inflammation combined with massive volume resuscitation cause increased IAP in these patients. IAP above 20 mmHg is common in patients with burns > 40% of their total body surface area, particularly if the patient is exposed to inhalational injury, poorly guided fluid resuscitation therapy or abdominal wall burns [25].

*Severe acute pancreatitis *is associated with IAH/ACS [6,26], with IAH occurring in 59% to 78% and ACS in 27% to 56% of these patients [27-29]. The combination of both primary (due to abdominal collections and inflammation) and secondary (due to fluid resuscitation) IAH is often observed, and this explains the high incidence of IAH/ACS in severe acute pancreatitis.

The term ACS was introduced by Kron et al. in 1984, who reported ACS after aortoiliac surgery [8]. Since then, the syndrome has been repeatedly described after *ruptured abdominal aortic aneurysm *(rAAA) repair [30-32]. Although only few population-based studies have specifically addressed rAAA, it is reasonable to count IAH/ACS as an important complication in these patients. Therefore, IAP monitoring after surgical or endovascular repair of rAAA is strongly recommended [33,34].

While IAH/ACS frequently occurs after *major abdominal surgery*, its true incidence is difficult to establish, as earlier studies use different definitions for IAH and do not always differentiate between IAH and ACS. Defining IAH as IAP ≥ 25 mmHg, Biancofiore et al. found a 32% incidence in liver transplant patients [35]. Sugrue et al. reported that 46% of emergency gastrointestinal surgery patients requiring intensive care had IAP ≥ 18 mmHg [36].

There are no clear data on the incidence and risk factors of IAH in *spontaneously breathing patients*. To our knowledge, the only study that included exclusively spontaneously breathing patients is by Serpytis and Ivaskevicius, who enrolled 77 non-infectious patients after major abdominal surgery [37]. They observed a high incidence of IAH (45.5% on the first postoperative day) and a positive correlation between positive fluid balance and IAP. The bladder pressure was measured with a 50 ml instillation volume, while the patients were asked to relax. This instillation volume may result in overestimation of IAP in mechanically ventilated and sedated patients [38-40], but it is not known how it may influence the results in spontaneously breathing patients. In studies on consecutive patients, especially when enrolling only the patients hospitalised for at least 24 h in the ICU, the majority of patients would expectedly be mechanically ventilated. In the prevalence study by Malbrain et al., 63% of patients with IAH and 70% of patients without IAH were on mechanical ventilation. IAH patients had a lower pO_2_/FiO_2 _ratio, but ventilation groups were not separately analysed [4]. The only study of consecutive ICU patients where mechanical ventilation was identified as a risk factor of IAH is the report by Vidal et al. [12]. In that study, 75% of IAH patients compared to 37% of non-IAH patients were mechanically ventilated. Other studies either do not report the proportion of MV [13,17,34,39] or enrolled only MV patients based on pathophysiological arguments of thoraco-abdominal interaction [14-16]. Thus, studies on the importance of IAH as well as IAP monitoring methods in spontaneously breathing intensive/intermediate care patients are warranted. Considering the major changes in physiological and pathophysiological conditions associated with mechanical ventilation, we caution against the free transmission of evidence between these two patient groups.

Because IAH has mainly been studied in mixed or surgical ICU populations, the data on *medical patients *are scarce. Malbrain et al. reported no difference in the prevalence of IAH between the patients of medical (54.4%) and surgical (45.6%) profile [3], no difference in the incidence of IAH was observed in the second study by the same authors [4]. In a study by Reintam et al., almost all patients with primary IAH and approximately 50% of patients with secondary IAH were of surgical profile [5]. Worse outcomes of secondary compared with primary IAH were observed. Daugherty et al. studied 40 medical patients with a minimum net positive fluid balance of 5 L/24 h [41]. In this small group, equal to 8.5% of all medical ICU admissions screened, the incidence of IAH was 85% and of ACS 33%. Positive fluid balance was also identified as an independent risk factor for IAH in several studies [4,12,13], whereas some other studies with mixed ICU patients failed to observe positive fluid balance as an independent risk factor for IAH [3,15]. Although the evidence is not entirely consistent and the cut-off value for positive fluid balance is not clear, routine IAP monitoring should be considered in medical patients with large-volume resuscitation.

Limited information is available about the prevalence of IAH in *septic patients*. Regueira et al. reported that 76.5% of their septic shock patients exhibited IAH during the first 72 h after admission [2]. Surprisingly many (27/67 (40%)) septic shock patients with IAH developed ACS. Other studies have demonstrated that the proportion of septic patients among IAH patients is higher than in the total ICU population [4,15]. The simultaneous presence of other factors, such as intra-abdominal infections, massive fluid resuscitation and ileus, may explain the high prevalence of IAH in this subgroup of patients.

### In which patients should IAP be measured?

Many literature reviews list all clinical conditions that have been associated with increased IAP and mistakenly call them possible risk factors of IAH [7,42]. Such an overwhelming list of all possible clinical conditions, including sepsis, oliguria, acidosis and others, would be of little help to an attending clinician to identify patients who would benefit from IAP assessment. Measuring IAP requires labour and money; therefore, its blind application in all patients should be avoided. From a critical analysis of the prevalence and risk factors above, the following summary can be made. More than one study has identified high BMI, abdominal surgery, liver dysfunction/ascites, hypotension/vasoactive therapy, respiratory failure and excessive fluid balance as risk factors of IAH in general ICU populations (Table 1). Importantly, recent studies that have incorporated IAH prevention may have identified different risk factors compared to earlier studies. To create a uniform and explicit list of independent risk factors applying to all ICU patients will probably be impossible. Different approaches seem unavoidable for different patient groups. Epidemiologic studies, analysing different groups only after including all consecutive patients, should be encouraged. We need to be careful when drawing conclusions about the incidence or risk factors of IAH based on studies including only selected patients.

At present, we suggest IAP be measured routinely in certain patient groups. Given the high risk of ACS, monitoring IAP is mandatory in ICU patients with severe burns, severe trauma, severe acute pancreatitis, liver failure or ruptured aortic aneurysms. Increased IAP in these patients is related to (1) decreased abdominal wall compliance (laparotomy, high BMI, respiratory mechanics and abdominal eschar); (2) increased intra-abdominal volume (ascites and intra-abdominal bleeding); (3) increased intraluminal volume (ileus, gastrointestinal bleeding and bowel oedema); or (4) capillary leaking and fluid overload (pancreatitis and shock/sepsis). IAP measurements are strongly indicated if the patient has any of these conditions.

Some evidence suggests [15] that the risk for IAH is minimal in mechanically ventilated ICU patients without pancreatitis, acute hepatic failure/cirrhosis with ascites, gastrointestinal bleeding or laparotomy and in the absence of BMI > 30, positive end-expiratory pressure > 10 cmH_2_O, PaO_2_/FiO_2 _< 300 and the use of vasopressors/inotropes on admission day. This list might be advocated to identify those ICU patients in whom IAP measurement can be forgone. It needs to be underlined that this evidence originates from one single-centre study in ventilated patients only, and most likely, this list would be different in different ICUs. However, similar local checklist might be the first step in decision-making process for IAP monitoring. The complexity of abdomino-thoracic interactions and the pathophysiology of polycompartment syndrome should always be kept in mind, as should the probable necessity of measuring IAP in each individual patient [43-46]. In addition to the standardised IAP measurements in at-risk patients, a routine clinical assessment of the signs of IAH should be a part of every patient's bedside evaluation, leading to prompt IAP monitoring in case of the slightest suspicion of (the development of) IAH. The detection of IAH should lead to the complex management of the syndrome, for which the management guidelines from WSACS provide good support [47-49].

## Conclusions

Clear guidelines to select the patients in whom IAP should be measured cannot be given at present. Based on the admission diagnosis, the authors strongly support IAP monitoring in mechanically ventilated patients with severe burns, severe trauma, severe acute pancreatitis, liver failure or ruptured aortic aneurysm. Ventilated and spontaneously breathing patients should be assessed differently, but no suggestions can be made regarding IAP monitoring in spontaneously breathing patients. The ICU patients in whom IAP measurements are not initiated on admission should undergo careful bedside evaluation with a low trigger for starting IAP monitoring. Further research is needed to identify the exact cohort of patients in whom IAP measurement is mandatory.

## Abbreviations

ACS: abdominal compartment syndrome; APACHE II: Acute Physiology and Chronic Health Evaluation II score; BMI: body-mass index; FiO_2_: the fraction of inspired oxygen; IAH: intra-abdominal hypertension; IAP: intra-abdominal pressure; ICU: intensive care unit; MV: mechanical ventilation; PaO_2_: partial pressure of oxygen in arterial blood; rAAA: ruptured abdominal aortic aneurysm; WSACS: World Society of Abdominal Compartment Syndrome.

## Competing interests

The authors declare that they have no competing interests.

## Authors' contributions

JS conceived the review, performed the literature search and drafted the manuscript. KT and ARB performed the literature search and participated in writing the manuscript. All authors have read and approved the final manuscript.
